# Autoregulation and dual stepping mode of MYA2, an *Arabidopsis* myosin XI responsible for cytoplasmic streaming

**DOI:** 10.1038/s41598-022-07047-0

**Published:** 2022-02-24

**Authors:** Takeshi Haraguchi, Kohji Ito, Takamitsu Morikawa, Kohei Yoshimura, Nao Shoji, Atsushi Kimura, Mitsuhiro Iwaki, Motoki Tominaga

**Affiliations:** 1 Department of Biology, Graduate School of Science, Chiba University, Chiba, 263-8522 Japan; 2 RIKEN Center for Biosystems Dynamics Research, RIKEN, Osaka, Japan; 3 Faculty of Education and Integrated Arts and Sciences, Waseda University, 2-2 Wakamatsu-cho, Shinjuku-ku, Tokyo, 162-8480 Japan; 4 Major in Integrative Bioscience and Biomedical Engineering, Graduate School of Science and Engineering, Waseda University, 2-2 Wakamatsu-cho, Shinjuku-ku, Tokyo, 162-8480 Japan

**Keywords:** Biophysics, Molecular biology, Plant sciences

## Abstract

*Arabidopsis thaliana* has 13 genes belonging to the myosin XI family. Myosin XI-2 (MYA2) plays a major role in the generation of cytoplasmic streaming in *Arabidopsis* cells. In this study, we investigated the molecular properties of MYA2 expressed by the baculovirus transfer system. Actin-activated ATPase activity and in vitro motility assays revealed that activity of MYA2 was regulated by the globular tail domain (GTD). When the GTD is not bound to the cargo, the GTD inhibits ADP dissociation from the motor domain. Optical nanometry of single MYA2 molecules, combining total internal reflection fluorescence microscopy (TIRFM) and the fluorescence imaging with one-nanometer accuracy (FIONA) method, revealed that the MYA2 processively moved on actin with three different step sizes: − 28 nm, 29 nm, and 60 nm, at low ATP concentrations. This result indicates that MYA2 uses two different stepping modes; hand-over-hand and inchworm-like. Force measurement using optical trapping showed the stall force of MYA2 was 0.85 pN, which was less than half that of myosin V (2–3 pN). These results indicated that MYA2 has different transport properties from that of the myosin V responsible for vesicle transport in animal cells. Such properties may enable multiple myosin XIs to transport organelles quickly and smoothly, for the generation of cytoplasmic streaming in plant cells.

## Introduction

Myosin is a motor protein that converts the chemical energy liberated by ATP hydrolysis into directed movement along actin filaments. Phylogenetic analyses of myosin sequences have shown that there are at least 79 myosin classes in eukaryotes^1^. The myosin superfamily shares a common domain composition: a conserved motor domain (MD) with ATPase and actin-binding activities, a neck domain comprising one to six repeats of isoleucine–glutamine (IQ) motifs acting as a lever arm, a coiled-coil domain, and a globular tail domain (GTD) that binds the cargo^2^. Many myosins of different classes participate in transport of various cargos, such as organelles, vesicles, and ribonucleoproteins, within cells^3^. Myosins bind different cargos via the GTD and transport them along actin filaments by the MD activity. Proper delivery and regulation of intracellular transport by myosins within cells is crucial for maintaining cellular function. The myosin motility is finely regulated by various mechanisms such as phosphorylation, calcium ions, and autoinhibition^4,5^. It has been reported that the activity of myosin V, myosin VIIA, and myosin X is regulated by autoinhibition using the GTD. In these myosins, the MD is typically maintained in an OFF state when the GTD is folded over and interacts with the MD to inhibit ATPase activity. Regulation by autoinhibition could avoid unproductive movements on actin filaments without cargo and wasteful ATP consumption. Conserved amino acid residues on both the MD and the GTD of myosin V mediate this interaction^6–8^.

Cytoplasmic streaming is the long-range, rotational movement of cytoplasm, widely observed in cells of organisms ranging from algae to angiosperms^9^. Cytoplasmic streaming is generated by organelle-associated plant-specific class XI myosin sliding along actin filaments^10^. Myosin XI has a typical myosin structure, consisting of an N-terminal MD, a neck domain comprising six tandem repeats of IQ motifs, a long coiled-coil domain for dimerization, and a C-terminal GTD that binds cargo. The molecular mechanism of myosin XI that generates fast cytoplasmic streaming has been investigated using tobacco 175 kDa myosin XI purified from cultured tobacco BY-2 cells. Tobacco 175 kDa myosin XI moves processively along actin filaments with approximately 35 nm steps^11^. The processive stepping motion is analogous to that of myosin Va, which is involved in intracellular transport in mammalian cells and moves on actin filaments in a “hand-over-hand” fashion^2^. Although the structural and motile features of tobacco 175 kDa myosin XI were similar to those of myosin Va, the *V*_max_ value of the velocity and of the ATPase activity of tobacco 175 kDa myosin XI were 4.6 µm s^−1^ and 76 s^−1^, respectively, which are about tenfold higher than those of myosin Va^11^.

Genome analysis revealed that *Arabidopsis thaliana* possesses 13 myosin XI members^12^. Gene knockout studies have revealed that myosin XI-1, XI-2, XI-B, XI-I, and XI-K are responsible for the movement of organelles, such as the endoplasmic reticulum, Golgi stacks, peroxisomes, and mitochondria^13–17^, concomitant with growth defects^15,16,18^. The full-length cDNAs and promoter regions for all 13 *Arabidopsis* myosin XIs have been cloned, and their tissue-specific expression and motile and enzymatic activities were identified^19^. The velocities and ATPase activities of the 13 *Arabidopsis* myosin XIs are significantly different, and the myosins are classified broadly into three groups—high, medium, and low—based on velocity. The velocity grouping appears to be roughly correlated with the tissue-specific expression patterns^19^. Gene knockout analysis indicated that *Arabidopsis* myosin XI-2 (MYA2) and XI-K are the major motor proteins that provide the motive force for cytoplasmic streaming^16,17^. Among 13 *Arabidopsis* myosin XIs, MYA2 is the closest to tobacco 175 kDa myosin XI phylogenetically. It is unclear whether the motile mechanisms of MYA2 is the same as that of tobacco 175 kDa myosin XI. In this study, the in vitro enzymatic properties and single-molecule motility of recombinant *Arabidopsis* MYA2 were analyzed. We found that MYA2 has different motile and regulatory properties than those of tobacco 175 kDa myosin XI.

## Results

### Constructs

**Figure 1:**
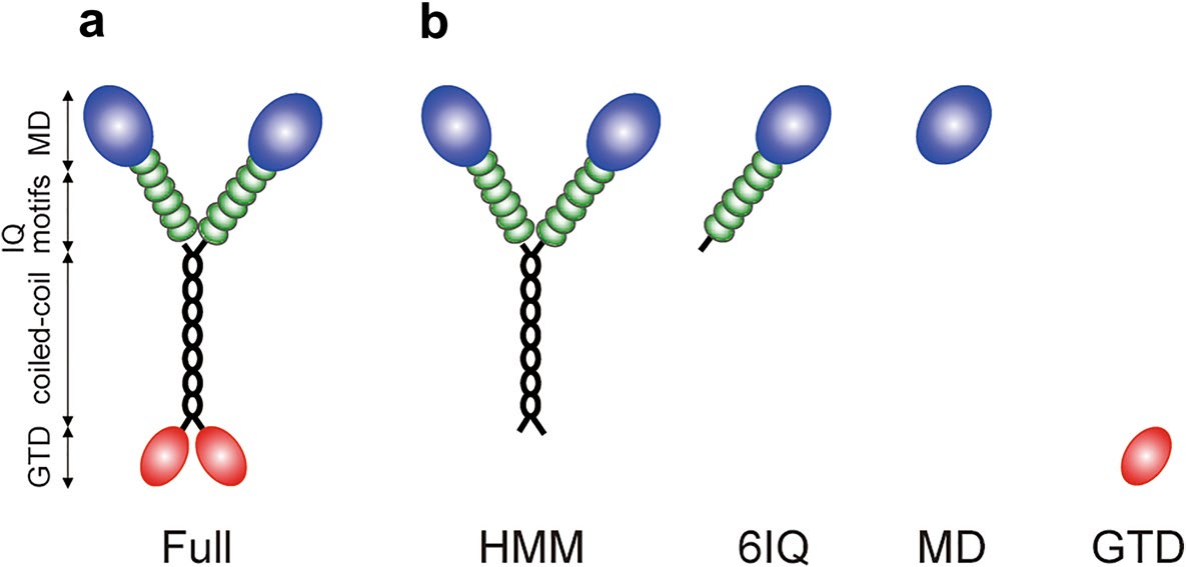
Schematic diagrams of the MYA2 constructs used in this work. (**a**) Full: Full-length construct comprising MD, six IQ motifs to which six calmodulins bind, coiled-coil, and GTD domains. (**b**) HMM: HMM construct comprising of MD, six IQ motifs to which six calmodulins bind, and a coiled-coil domain. 6IQ: 6IQ construct comprising of MD and six IQ motifs to which six calmodulins bind. MD: MD construct comprising only MD. GTD: GTD construct comprising only GTD.

A schematic diagram of full-length MYA2 (Full) deduced from its amino acid sequence is shown in Fig. 1a. In general, the domain presence and composition were consistent across all 13 *Arabidopsis* myosin XIs^20^. *Arabidopsis* myosin XI is composed of a MD, a neck domain with six IQ motifs to which calmodulin or calmodulin-like proteins bind, a coiled-coil region, and a GTD (Fig. 1a)^9,19,20^. In this study, we generated five types of recombinant MYA2 constructs: Full (Fig. 1a); heavy meromyosin which lacked GTD (HMM); motor domain with six IQ motifs (6IQ); MD; and GTD (Fig. 1b)^19^. These constructs were expressed using a baculovirus transfer system in High Five insect cells. The recombinant MYA2 proteins were purified using both nickel-affinity and Flag-affinity resin, as previously reported^19,21^. SDS-PAGE analysis was performed to confirm the purity and homogeneity of the proteins (Fig. S1a), and binding of calmodulins to the purified 6IQ (Fig. S1b).

### Inhibitory effects of GTD on actin-activated ATPase activities

**Figure 2:**
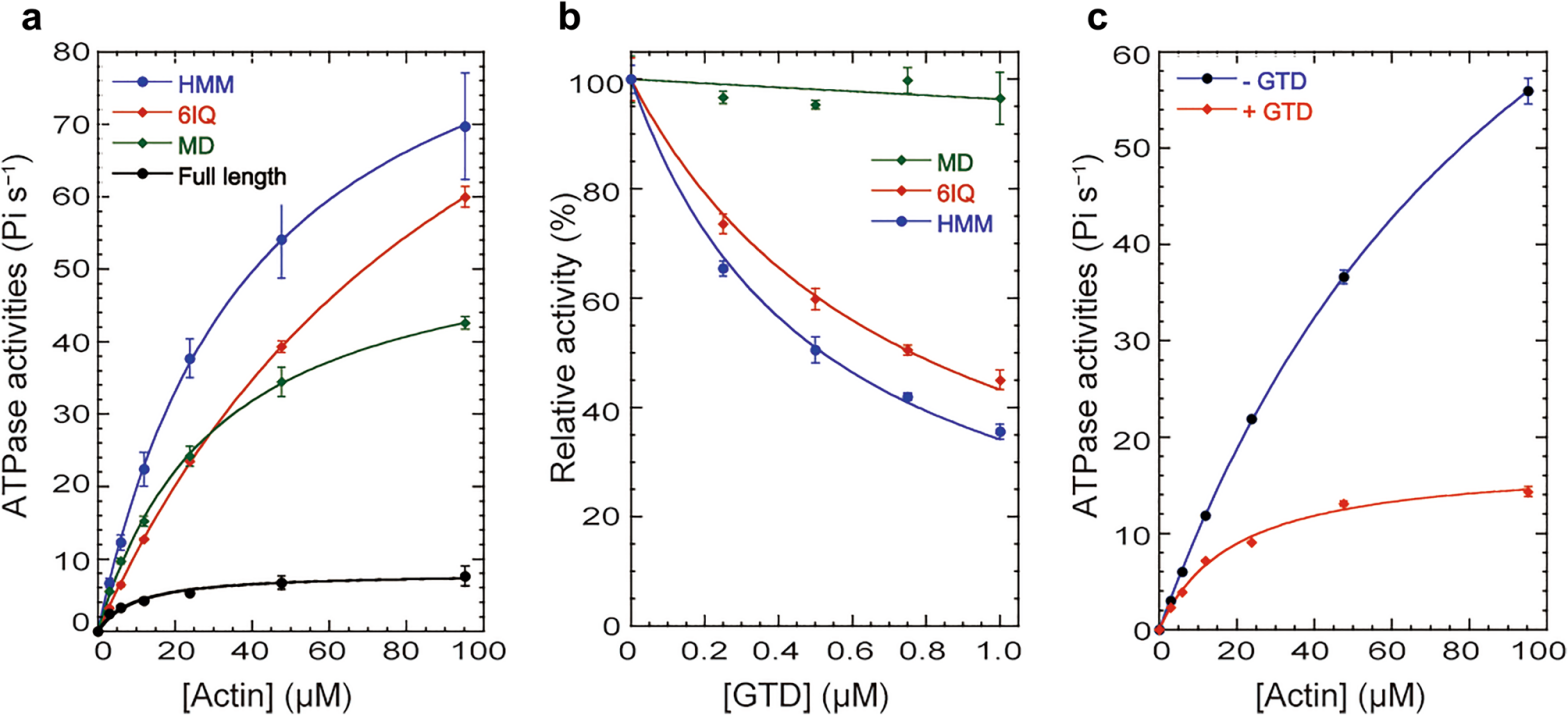
Actin-activated Mg^2+^-ATPase activities and inhibitory effects on Mg^2+^-ATPase activities of Full, HMM, 6IQ, and MD. (**a**) Actin-activated Mg^2+^-ATPase activities of Full, HMM, 6IQ, and MD. Mg^2+^-ATPase activities in the presence of various actin concentrations. The data were fitted using Michaelis–Menten dynamics, and the *V*_max_ and K_app_ values were measured. The concentrations of Full, HMM, 6IQ, and MD used 0.005–0.019, 0.001–0.009, 0.002–0.018, and 0.001–0.008 µM, respectively. (**b**) Inhibitory effects of GTD on Mg^2+^-ATPase activities of HMM, MD, and 6IQ. The actin-activated ATPase activities of HMM and 6IQ in the presence of various concentrations of GTD and 23.8 μM actin were fit to a hyperbola, and the *K*_i_ values of GTD for HMM and 6IQ were calculated to be 0.5 μM and 0.8 μM, respectively. (**c**) Inhibitory effects of Mg^2+^-ATPase activities of 6IQ by 1 µM GTD in the presence of various actin concentrations.

Figure 2a shows the actin-activated ATPase activities of MYA2 constructs at various actin concentrations. Plots of ATPase activity against actin concentration were found to fit a Michaelis–Menten type curve. Using this curve, we determined the maximum rate of ATP turnover (*V*_max_) and the actin concentration at which the ATPase rate reached half of its maximum (*K*_m_). The *V*_max_ values of HMM, 6IQ, and MD were 99 s^−1^, 127 s^−1^, and 56 s^−1^^19^, respectively (Fig. 2a). In contrast, the *V*_max_ value of Full was 8.1 s^−1^ much smaller than those of HMM, 6IQ, and MD (Fig. 2a). The low *V*_max_ value of Full was likely due to the inhibitory effect of the GTD on actin-activated ATPase activity. To confirm inhibition by the GTD, the actin-activated ATPase activities of HMM, 6IQ, and MD were measured in the presence of various concentrations of GTD. The GTD inhibited the actin-activated ATPase activities of HMM and 6IQ in a concentration-dependent manner, but not that of MD (Fig. 2b).

The actin-activated ATPase activities of HMM and 6IQ in the presence of various concentrations of GTD and 23.8 μM actin were fit to a hyperbola, defining an inhibition constant (*K*_i_) of GTD. The *K*_i_ values of GTD for HMM and 6IQ were 0.5 µM and 0.8 µM, respectively (Fig. 2b). These results suggest that the GTD inhibits the actin-activated ATPase activities of MYA2, and that this inhibition requires IQ motifs. Figure 2c shows the actin-activated ATPase activities of 6IQ in the presence of 1 µM GTD and the absence of GTD. The *V*_max_ values of 6IQ in the absence and presence of GTD were 127 s^−1^ and 18 s^−1^, respectively. The *K*_m_ values of 6IQ for actin in the presence of 1 µM GTD and in the absence of GTD were 19 µM and 106 µM, respectively. At all actin concentrations, ATPase activities in the absence of the GTD were higher than ATPase activities in the presence of the GTD. The *V*_max_ value decreased by about seven times in the presence of 1 µM GTD.

**Figure 3:**
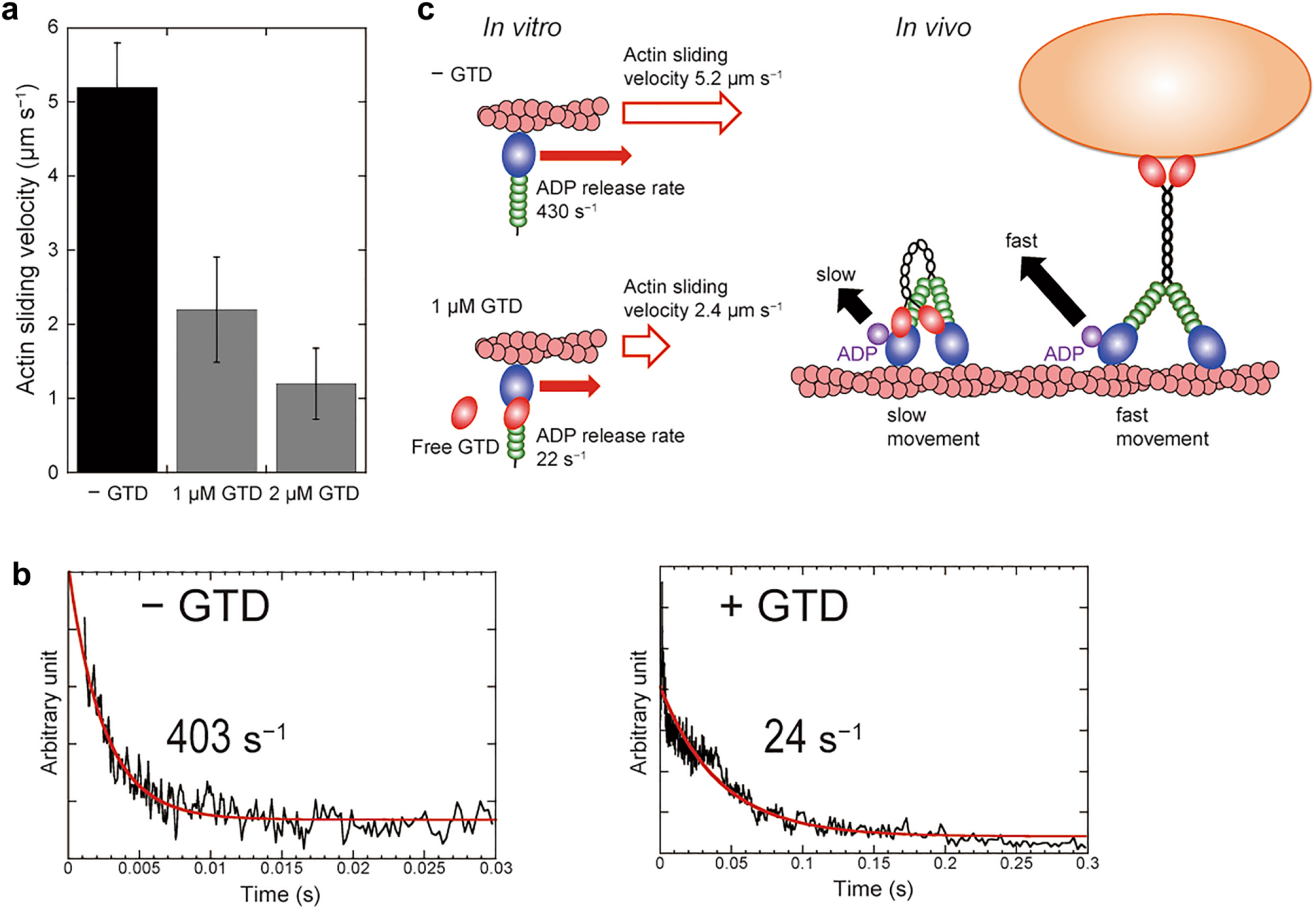
Inhibitory effects of actin sliding velocities of 6IQ and the dissociation rates of mant-ADP from 6IQ. (**a**) Inhibitory effects of actin sliding velocities of 6IQ by 1 µM GTD and 2 µM GTD in the presence of 3 mM ATP. (**b**) Mant-ADP dissociation from acto-6IQ in the presence of 1 µM GTD and absence of GTD. The dissociation rates of mant-ADP from 6IQ were measured using fluorescence energy transfer between the tryptophans of MD of 6IQ and mant-ADP. The transient shown is the average of six separate recordings. The red line is a single exponential fit yielding a rate constant of 403 s^−1^ in the absence of GTD (left) and 24 s^−1^ in the presence of 1 µM GTD (right). The average of three independent assays was 22 ± 3.4 s^−1^ in the presence of 1 µM GTD and 430 ± 40 s^−1^ in the absence of GTD. (c) A model of the mechanism by which GTD inhibits the movement of MYA2 in vitro and in vivo. In vitro, in the absence of GTD, the ADP dissociation rate of acto-6IQ is 430 s^−1^ and the actin sliding velocity of 6IQ is 5.2 μm s^−1^. In the presence of 1 μM GTD, the ADP dissociation rate of acto-6IQ is 22 s^−1^ and the actin sliding velocity of 6IQ is 2.4 μm s^−1^. In vivo, when GTD is not bound to the organelle, it interacts with MD and inhibits ADP dissociation from MD, thus slowing down the velocity of MYA2. In contrast, GTD is bound to the organelle, it does not interact with MD and thus does not inhibit MYA2 movement.

A similar inhibitory effect of GTD on the actin-activated ATPase activity has been reported for myosin V. Electron microscopy studies have shown that in the absence of cargo, full-length myosin V takes a folded-structure, in which the GTD bound to the head inhibits the actin-activated ATPase activity^22^. Because the inhibitory effects of GTD on the actin-activated ATPase activity and the myosin structure were similar between myosin V and MYA2, as shown above, the mechanism of inhibition by the GTD may be similar for the two myosins (Fig. 3c).

The inhibitory effect of GTD on the ATPase activity of full-length myosin V is released by Ca^2+^^23^. To investigate whether the Ca^2+^ releases the GTD-induced inhibitory effect of full-length MYA2, similar to that of myosin V, we measured the actin-activated Mg^2+^ ATPase activity of Full in the presence of Ca^2+^. The actin-activated ATPase activities of Full did not increase but decreased by 30% by adding 100 μM Ca^2+^ (Fig. S2a). This result shows that, unlike in the case of myosin Va, Ca^2+^ did not release the GTD-induced auto-inhibitory state of MYA2. The decrease in the activities by Ca^2+^ may be due to the dissociation of some of the calmodulin from IQ motifs.

### Inhibitory effects of GTD on motility and ADP dissociation from acto-myosin

To investigate the inhibitory effect of GTD on the MYA2 motility in an in vitro motility assay, we measured the actin sliding velocity by 6IQ in the presence of exogenous free GTD because the C-terminus of Full interacts with the glass surface, thus inhibiting the binding of the Full GTD to MD.

6IQ moved actin filaments at 5.2 µm s^−1^ in the presence of 3 mM ATP, which was similar to the value of Full^19^. In the presence of 1 µM GTD, the velocity dropped to less than half of that in the absence of the GTD (Fig. 3a). This result indicates that GTD inhibited the motility of MYA2 containing IQ motifs, as well as the actin-activated Mg^2+^ ATPase activity. For most myosins, including myosin XI, the actin sliding velocity is mainly determined by the ADP dissociation rate from acto-myosin^24–27^. The ADP dissociation rate from acto-6IQ in the presence and absence of the GTD was determined by measuring the decrease in the fluorescence intensity of mant-ADP using the stopped-flow apparatus^24–29^ as described in “Methods”. In the absence of GTD, the ADP dissociation rate from acto-6IQ was 430 ± 40 s^−1^. In the presence of 1 µM GTD, the rate was 22 ± 3.4 s^−1^, which is about 1/20 of that in the absence of GTD (Fig. 3b). These results suggest that free GTD, not bound to the cargo, inhibits the dissociation of ADP from MYA2 containing IQ motifs (Fig. 3c).

Then, we investigated whether Ca^2+^ releases the inhibitory effect of GTD on HMM motility. The velocity of HMM in the presence of 1 µM GTD did not increase but decreased by 30% by adding 100 μM Ca^2+^ (Fig. S2b). This result shows that Ca^2+^ did not release the GTD-induced inhibitory effect of MYA2, similar to the results of actin-activated ATPase activities as shown above.

### Processivity and stepping dynamics of MYA2

Next, we investigated the processivity of single MYA2 Full using TIRFM. Although MYA2 fused with GFP at the MD showed non-processive motion along an actin filament at a saturating concentration of ATP (1 mM ATP), it processively moved in one direction at a concentration of 1 µM ATP. The fluorescence of GFP showing processive motions photobleached in a single- or double-step, strongly suggesting single-molecule processivity (Fig. S3).

**Figure 4:**
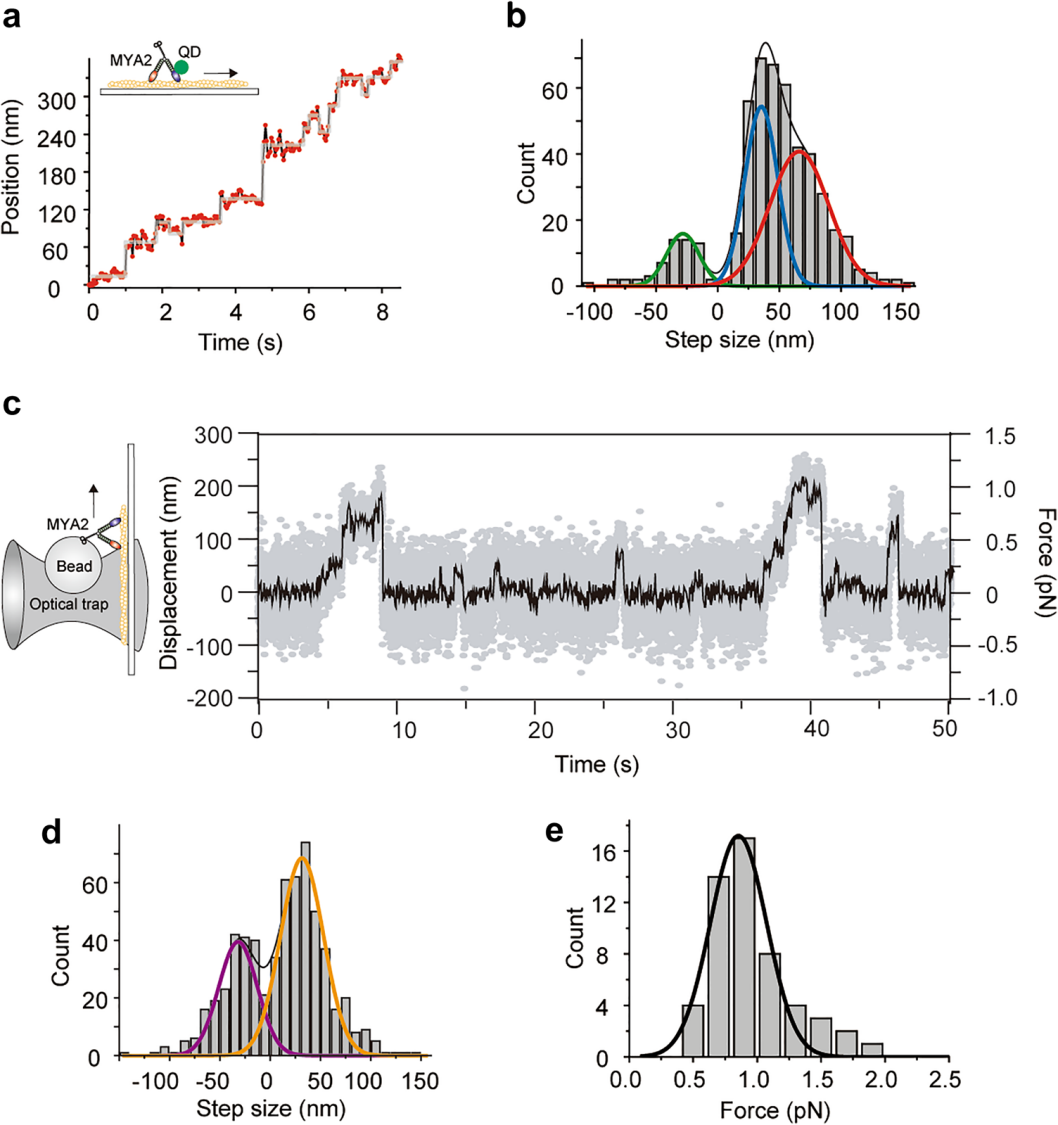
Stepping dynamics and force measurement of MYA2. (**a**) QD attached to a motor domain in a MYA2 dimer was observed using TIRFM at 2 nm and 33 ms spatiotemporal resolution. Steps were analyzed by an automated step-finding algorithm^43^ (gray line). ATP concentration, 1 µM. (**b**) Step size histogram of the motor domain. The histogram fit to three Gaussian functions with peaks at 60.5 ± 14.8 nm (red line), 29.3 ± 6.9 nm (blue line), and  − 28.1 ± 9.9 nm (green line), respectively. Black line indicates the convolution of three Gaussian functions. (**c**) Typical trace of optical trapping nanometry at 1 µM ATP. Gray line: raw trace; Black line: data passed through a 200 Hz low pass filter. (**d**) Step size histogram of an optically trapped bead attached to the MYA2 C-terminal tail. The histogram was fit to two Gaussian functions with peaks at 31.6 ± 15.2 nm (orange line), and − 31.9 ± 13.6 nm (purple line), respectively. Black line indicates the convolution of the two Gaussian functions. (**e**) Histogram of MYA2 stall force. The histogram was fitted to single Gaussian function with peaks at 0.85 ± 0.16 pN (black line).

Then, to dissect the stepping dynamics during the processive motion, we attached a streptavidin-conjugated fluorescent quantum dot (QD) to a biotinylated Halo-tag domain fused at the N-terminus of MD in a MYA2 dimer. We then tracked the motion with 2 nm precision and 33 ms time resolution using the FIONA method^30^ (Fig. 4a). We have previously measured the step sizes of myosin Va, which is known to move by a hand-over-hand mechanism, using the same method in this study and confirmed the step size distribution fit to a single Gaussian function with peak at 75 ± 9 nm (mean ± SD) (unpublished result). However, MYA2 showed broader forward step size distribution (Fig. 4b) similar to that of myosin VI which has large and small forward step sizes^31,32^. Therefore, we fit the step size distribution to a three Gaussian function with peaks at 60 nm (large forward steps), 29 nm (small forward steps) and − 28 nm (backward steps). To exclude the possibility that steric hindrance by the QD was responsible for the stepping dynamics, we also examined tetramethyl rhodamine (TMR)-labeled MYA2. The TMR was labelled at the same position as the QD (a Halo-tag domain fused at the N-terminus of MD). The velocity and run length of TMR-labeled MYA2 were similar to those for QD-labeled MYA2 (Table S1), and the three types of steps were also resolved for TMR-labeled MYA2 (Fig. S4). These results indicated that steric hindrance due to QD labeling at the MD was not significant. A histogram of the dwell time just before the forward steps was fit to a convolution of two exponentials (t*k*^2^exp[-*k*t]), in which *k* is a rate constant (Fig. S5). This is reasonable if we assume that QD-labeled and -nonlabelled MDs alternate steps, each corresponding to the same rate constant, *k*^30^.

**Figure 5:**
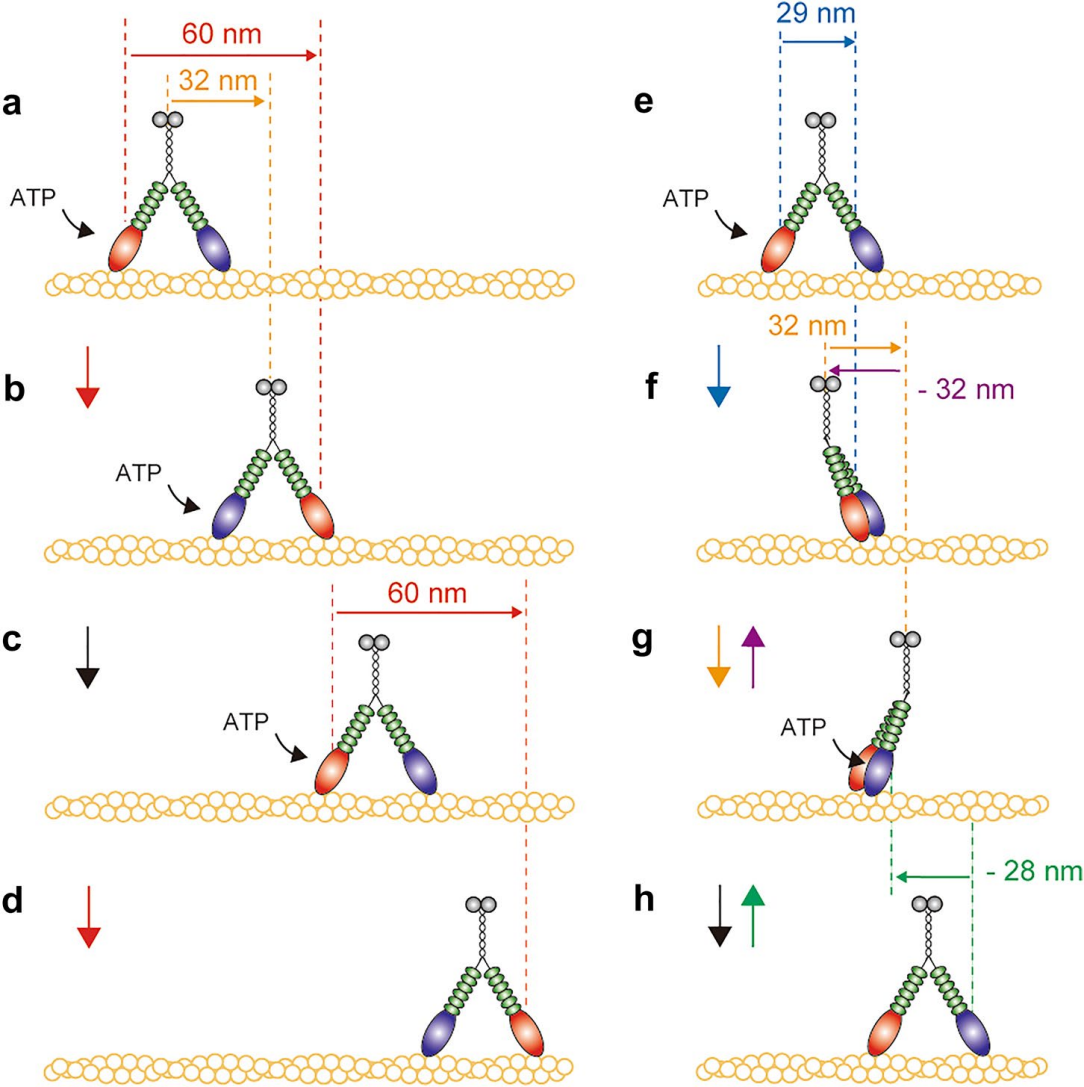
Walking model of MYA2. Left (**a**–**d**), large hand-over-hand steps; right (**e**–**h**), small inchworm-like steps. Colors denote the following: N-terminal motor domain (red or blue), lever arm (six calmodulins, green), coiled-coil domain (black), and the globular C-terminal tail domain (gray). Red, blue, and green arrows indicate the step of the MD for large, small, and backward steps, respectively, observed using FIONA. Orange and purple arrows indicate the step of the C-terminal tail for forward and backward steps, respectively, observed by optical trapping assay.

Three types of the step sizes (large and small forward steps, and backward steps) and the dwell time distribution just before the forward steps were consistent with those of myosin VI. In myosin VI, large and small forward steps can be explained by a hand-over-hand motion and inchworm-like motion, respectively^31^. Therefore, we concluded the large (60 nm) and small (29 nm) forward steps observed in MYA2 were caused by a hand-over-hand and inchworm-like motion, respectively (Fig. 5).

### Weak force generation of MYA2

To examine the force generation of MYA2, we attached a fluorescent polystyrene bead to the C-terminal tail and applied force using an optical trap. Figure 4c shows the typical trace of a single MYA2 Full under load at 1 µM ATP, and the step size histogram is shown in Fig. 4d. We used single-trap geometry and observed the bead displacement, which reported us the C-terminal tail displacements. The histogram was fit to two Gaussian functions with peaks at 32 nm (forward steps), and  − 32 nm (backward steps). Figure 4e shows the histogram of the stall force fit to a Gaussian function with peak at 0.85 ± 0.16 pN (mean ± SD).

## Discussion

In this study, we analyzed the in vitro enzymatic properties of MYA2, which is known to be a major force generator of cytoplasmic streaming in *Arabidopsis*^9^. The *V*_max_ value of the actin-activated ATPase activities measured using recombinant HMM and 6IQ, and the MD of MYA2 were higher than that of myosin V, and similar to that of tobacco 175 kDa myosin XI (Fig. 2a). This high ATPase activity is consistent with MYA2 acting as a driving force for cytoplasmic streaming. In contrast, the *V*_max_ value of the ATPase activity of MYA2 Full was much lower than those of HMM, 6IQ, and MD (Fig. 2a). This result indicated that the activity of MYA2 was inhibited by the GTD. Exogenous addition of the GTD inhibited the actin-activated ATPase activities of HMM and 6IQ in a concentration-dependent manner, but not that of MD, suggesting that GTD inhibits ATPase activity via the IQ motif (Fig. 2b). Such GTD suppression was not observed in full-length tobacco 175 kDa myosin XI purified from tobacco BY-2 cells^11^. Autoinhibition using GTD has been reported in myosin V, myosin VIIA, and myosin X. This intramolecular regulation could avoid unproductive movements on actin filaments without cargo and wasteful ATP consumption. The same intramolecular regulation in MYA2 suggests that MYA2 is not merely responsible for driving continuous cytoplasmic streaming, but also for more complex transport equipped with the regulatory system. The result that free GTD slows down ADP dissociation from acto-6IQ (Fig. 3b) indicates that MYA2 stays on actin filaments for a long time until the GTD binds to the cargo. This regulation may contribute to efficient intracellular transport, as in the following hypothesis. When MYA2 is not bound to cargo, it would pause on the actin filament while suppressing ATP consumption (Fig. 3c; slow movement in vivo). As soon as the GTD binds to a cargo, MYA2 can participate in the transport (Fig. S6). It has been shown that Ca^2+^ releases the head–tail interaction and the autoinhibition of myosin Va^23,33^, and that calmodulin bound to the first IQ motif of myosin Va is involved in head–tail interaction^34^. In contrast, Ca^2+^ did not release the autoinhibition of MYA2 (Fig. S2), suggesting that Ca^2+^ may not be related to the regulation of MYA2, unlike myosin V. We confirmed that calmodulin was bound to the IQ motifs of the purified 6IQ (Fig. S1). However, it was impossible to quantify the molar ratio of calmodulin bound to MYA2 because the amount of purified 6IQ was small and the molecular weight of calmodulin is much smaller than 6IQ, leading to the thin calmodulin band.

The duty ratio of myosin is defined as the ratio of time spent strongly bound to actin to the cycle time of actin-activated ATPase. The time spent strongly bound to actin is the sum of the acto-myosin-ADP (AM.ADP) and acto-myosin (AM) states^35^. Because the AM state is very short for most myosins, the time spent strongly bound to actin is almost equal to that spent in the AM.ADP state, which is approximated by the reciprocal of the ADP dissociation rate from acto-6IQ (1/403 s^−1^ = 2.48 ms). The cycle time of actin-activated ATPase is approximated by the reciprocal of the *V*_max_ value of 6IQ and is 7.87 ms. Thus, the duty ratio of MYA2 is expected to be 32% (100 × 2.48 ms/7.87 ms = 32%), showing that MYA2 is a non-processive myosin.

The single-molecule motility of MYA2 revealed its unique motility, which is quite different from that previously reported for tobacco 175 kDa myosin XI^11^. Optical nanometry of single MYA2 molecules using TIRFM revealed that MYA2 was non-processive at high ATP concentrations, which is consistent with the duty ratio estimation. However, MYA2 demonstrated processive motion on an actin filament at low ATP concentrations. FIONA revealed that one MD in a MYA2 dimer moved with three different step sizes: − 28 nm, 29 nm, and 60 nm, and an optical trapping assay revealed the C-terminal tail moved with ± 32 nm step sizes. Based on this observation, we propose the model for MYA2 step movement shown in Fig. 5. Forward large steps (60 nm) are explained by a hand-over-hand mechanism in Fig. 5a–d. First, the trailing MD unbinds from actin upon ATP binding, moves forward and binds to the forward actin target 60 nm ahead (Fig. 5a,b). Second, the new trailing MD moves forward in the same manner (Fig. 5b–d). Because the QD was labeled in one MD of a dimer, two ATPase cycles were required before our observation of the 60 nm forward steps. This is consistent with the dwell time distribution explained by a convolution of two exponentials (Fig. S5). The hand-over-hand mechanism shows the C-terminal tail moves forward 32 nm as observed by an optical trapping assay (Fig. 4d). Forward small steps (29 nm) are shown in Fig. 5e–h by an inchworm-like mechanism. Here, the trailing MD unbinds from actin upon ATP binding and binds to the forward actin target 29 nm ahead (Fig. 5e,f). Then, the two lever arms tilt and the C-terminal tail moves forward 32 nm (Fig. 5f,g). Finally, the leading MD undergoes a small forward step (Fig. 5g,h) to return to Fig. 5e. state. For backward steps of − 28 nm of MD, the reversal motion from Fig. 5f to e or from Fig. 5h to g is probable. However, the lever arm swing (Fig. 5f,g) should be a fast transition, therefore, the reversal motion from Fig. 5f to e is not considered in our model. Such a reversal motion is inhibited in myosin VI stepping^31^. For backward steps of − 32 nm by the C-terminal tail, a reversal motion by the hand-over-hand mechanism is inhibited because − 60 nm steps of MD were not observed. Instead, the most probable reversal motion of the lever arm swing is an inchworm-like mechanism (from Fig. 5g to f).

Force measurement using optical trapping showed that the stall force of MYA2 was 0.85 pN, which was similar to that of tobacco 175 kDa myosin XI (0.5 pN) and less than half that of myosin V (2–3 pN)^36,37^. These results from single molecular analysis suggested that MYA2 properly uses dual stepping mode by hand-over-hand and inchworm mechanism in vivo. Proper use of dual stepping mode may be advantageous in avoiding interference between myosin molecules when many motors are closely associated on the same organelles. That is, if a certain myosin interferes with the myosin in front of it, it is possible to briefly pause the movement of the trailing myosin by its stepping back a small half step, without large backstepping. MYA2 may be a suitable motor for driving large organelles, such as the ER with multiple molecules simultaneously. Since the organelle carried by MYA2 and the number of MYA2 molecules working on the organelle have not been clarified, further cell biological analysis is required.

Tobacco 175 kDa myosin XI was phylogenetically the closest to MYA2 among the 13 *Arabidopsis* myosin XI members. However, considerable differences were found in the mode of movement (Table S2) and in their regulation, suggesting that myosin XIs have diverse molecular functions.

## Methods

### Protein engineering and expression

A baculovirus transfer vector for pFastBacMYA2 was generated as follows. MYA2 (AGI code: AT5g43900.1, UNIPROT ID: Q9LKB9) cDNA cloned from *Arabidopsis* seedlings was mutated to create an NcoI site at the upstream region of the nucleotide sequence encoding residue 1, and an AgeI site in the downstream region of the nucleotide sequence encoding residue 4515 of MYA2. These sequences were cut with NcoI and AgeI and the fragment was ligated with the NcoI-AgeI fragment of the pFastBac MD with a FLAG-tag^27^. The resulting construct, MYA2, encodes the N-terminal sequence (MSYYHHHHHHDYKDDDDKNIPTTENLYFQGA) containing a His_6_-tag and a FLAG-tag (DYKDDDDK), residues 1–1505 of MYA2, a flexible linker (GGG), a Myc-epitope sequence (EQKLISEEDL), and a His_8_-tag. A baculovirus transfer vector for pFastBac Halo-tag-fused MYA2 was generated as follows. The pFN21A (HaloTag (R) 7) CMV Flexi (R) Vector (Promega Corporation, Madison, USA) was mutated to create an NcoI site downstream of the nucleotide sequence encoding the HaloTag 7 open reading frame and was cut with NcoI. The NcoI digestion fragment was ligated with the NcoI digestion fragment of pFastBacMYA2. The resultant construct, Halo-tag-fused MYA2, encodes the N-terminal sequence (MSYYHHHHHHDYKDDDDKNIPTTENLYFQGA) containing a His_6_-tag, a FLAG-tag (DYKDDDDK), and a Halo-tag 7, residues 1–1505 of MYA2, a flexible linker (GGG), a Myc-epitope sequence (EQKLISEEDL), and a His_8_-tag. A baculovirus transfer vector for pFastBac *Arabidopsis* calmodulin was generated as follows. *Arabidopsis* calmodulin (AT3G56800.1) cDNA cloned from *Arabidopsis* seedlings was mutated to create an XbaI site upstream of the nucleotide sequence encoding residue 1, and an XhoI site downstream of the nucleotide sequence encoding residue 448 of *Arabidopsis* calmodulin. The XbaI-XhoI digestion fragment was ligated with the XbaI-XhoI digestion fragment of pFastBac 1 (Invitrogen, Carlsbad, CA, USA). To express MYA2 and Halo-tag fused MYA2, 800 mL of High Five TM cell culture (Invitrogen) was infected with viruses expressing the respective constructs. *Arabidopsis* calmodulin was co-produced by co-infection with a virus expressing *Arabidopsis* calmodulin. The infected cells were cultured in Erlenmeyer flasks (1 L × 4) at 28 °C and shaken at 130 rpm, for 43 h. The study complies with national guidelines in Japan.

### Protein purification

Protein purification was performed as previously described with some modifications^35^. Cells were harvested and washed with 150 mM NaCl, 1 mM EGTA, and 10 mM HEPES, pH 7.4. The pelleted cells were suspended with 2 vol/g cells of buffer A (30 mM HEPES, pH 7.4, 200 mM KCl, 5 mM MgCl_2_, 4 mM ATP, 1 mM EGTA, 1 mM DTT, and a mixture of protease inhibitors). Then, 2 vol/g cells of buffer A containing 2% Nonidet P-40 was added and mixed. After incubation on ice for 10 min, the lysate was centrifuged at 228,000×*g* for 30 min. The supernatant was mixed with 0.3 mL of anti-FLAG M2 affinity resin (Sigma-Aldrich, St. Louis, MO, USA) in a 50 mL tube on a rotating wheel for one hour at 4 °C. The resin suspension was then loaded on a column and washed with 30 mL of buffer A containing 1 µM mouse calmodulin. Halo-tag-fused MYA2 was biotinylated by incubation with buffer A containing 10 µM of HaloTag PEG-Biotin Ligand (Promega) in the column for 10 min. MYA2 and Halo-tag-fused MYA2 was eluted with buffer A containing 0.2 mg/mL of 3× FLAG peptide (Sigma-Aldrich). *Arabidopsis* calmodulin was expressed in High Five TM cells by infecting them with virus expressing *Arabidopsis* calmodulin and purified using the method of Awata et al.^38^. *Gallus gallus* skeletal muscle actin was prepared using method of Spudich and Watt^39^.

### ATPase activity

ATPase activities were determined by measuring released phosphate as previously described^40^. The reaction mixtures for the assay of actin-activated Mg^2+^-ATPase activity contained 25 mM KCl, 4 mM MgCl_2_, 25 mM HEPES–KOH (pH 7.4), 3 mM ATP, 1 mM DTT, 1 mg/mL BSA, and, at 25 °C, 0.125—4 mg/mL F-actin.

### In vitro gliding assay

The velocity was measured using an anti-myc antibody-based version of the in vitro actin filament gliding assay as previously described^27^. The velocity of actin filaments was measured in 150 mM KCl, 4 mM MgCl_2_, 25 mM HEPES–KOH (pH 7.4), 2 mM ATP, 10 mM DTT, and oxygen scavenger system (120 µg/mL glucose oxidase, 12.8 mM glucose, and 20 µg/mL catalase) at 25 °C. Average sliding velocities were determined by measuring the displacements of actin filaments.

### Measurement of ADP dissociation rate using a stopped-flow apparatus

Experiments were done in 25 mM KCl, 4 mM MgCl_2_, 1 mM DTT, and 25 mM Hepes pH 7.4, at 25 °C, using an Applied Photophysics SX18MV stopped-flow spectrophotometer (dead time: 1.15 ms). The mixture of 0.4 µM 6 IQ, 60 µM mant-ADP, 20 µM actin in the presence (1.6 µM) or absence of GTD was mixed with 3 mM ATP (each concentration is the concentration after mixing). Mant-ADP were excited at 290 nm via fluorescence resonance energy transfer from tryptophan of MD, and emission was observed after passing through a 389 nm cutoff filter. Dissociation of mant-ADP from was monitored by the decrease of its fluorescence.

### Single-molecule imaging

Single-molecule imaging was performed as previously described^31^. Briefly, the Halo-tag domain fused at the N-terminus of MD was attached with HaloTag PEG-Biotin Ligand (G859A, Promega) or HaloTag TMR Ligand (G825A, Promega) during the purification of MYA2. Then, streptavidin-conjugated QD (Qdot585, Q10113MP (Invitrogen)) and biotinylated-MYA2 were mixed at a molar ratio of 1:1 and incubated overnight on ice. Assay buffer (AB; 30 mM HEPES–KOH [pH 7.8], 25 mM KCl, 5 mM MgCl_2_, and 2 mM EGTA) was prepared before each experiment. Sample chambers were assembled using double-sided transparent tape (Scotch) and dried coverslips. Actin filaments were quickly flowed into sample chambers in which α-actinin molecules were directly adhered to a glass slide. The glass surface was then coated with 5 mg/mL casein. A 1:1 QD–MYA2 mixture was diluted 100 times in motility buffer (MB; AB plus, 1 µM ATP, 0.2 mg/mL glucose oxidase, 4.5 mg/mL glucose, 36 mg/mL catalase, 1% v/v 2-mercaptoethanol^41^, 2 mM phosphocreatine, 0.1 mg/mL creatine phosphokinase^32^, 0.1 mg/mL calmodulin). This mixture was added into the sample chamber, and the chamber was sealed with nail polish and observed immediately. QD-conjugated MYA2 movement was imaged using TIRFM, and the excitation was provided by a 405 nm laser light (Compass405–50CW; Coherent, Santa Clara, CA, USA). The fluorescent photons were collected with a back-illuminated EMCCD camera (DV887ECS-BV; Andor Technology, Belfast, Northern Ireland). The fluorescence was passed through a dichroic mirror (DML557 nm; Asahi Spectra, Tokyo, Japan) and emission filter (FF01-593/40-25, Semrock, Rochester, NY, USA). Image acquisition was performed by commercial software (Andor, SOLIS software). The sample was kept at ambient temperature during data collection (27 ± 2 °C). Exported 8-bit data was analyzed by a program custom written in LabVIEW (National Instruments, Austin, TX, USA). The spot center for each frame was determined using a two-dimensional Gaussian fit according to a published method^30,42^. The accuracy of detection of the spot center was 2.0 nm. All steps were analyzed by an automated step-finding algorithm^43^.

### Optical trapping

Optical trapping was performed as previously described, with some modifications^44^. Briefly, carboxylate modified polystyrene beads (Invitrogen; 0.2 µm in diameter) were cross-linked to c-Myc monoclonal antibody (Clontech, Shiga, Japan) and coated with 10 mg/mL BSA. One microliter of the beads and c-myc tagged MYA2 were mixed and incubated for 30 min in AB containing 10 mg/mL BSA at a ratio of 1:20. The ratio was adjusted so that less than 10% of the beads displayed movement. Sample chambers were assembled using double-sided transparent tape (Scotch) and dried coverslips. Fluorescent dye-labeled actin filaments were quickly flowed into sample chambers in which α-actinin molecules were directly adhered to a glass slide. The glass surface was then coated with 5 mg/mL casein. One microliter of MYA2 coated beads was diluted 100 times in MB. This mixture was added to the sample chamber, and the chamber was sealed with nail polish and observed immediately. The optics of the optical trapping and detection of the bead positions were conducted as previously described^32,44^. Bead displacements were recorded at a sampling rate of 24 kHz with a bandwidth of 10 kHz. The load exerted on the beads was calculated from the bead displacement multiplied by the trap stiffness (~ 7.4 fN/nm), which was determined from the variance of the Brownian motion of a trapped bead by the equipartition theorem of energy. We determined the step sizes after passing the data through a low pass filter with a bandwidth of 200 Hz, using a custom program in DADiSP (CAE Solutions, Burlington, MA, USA) and Visual C++ (Microsoft, Redmond, WA, USA). All steps were checked by eye. To determine the precise step size at low loads, we calculated the elastic component in our single trapping geometry and estimated the correction factor depending on load^45^.

## Supplementary Information

Supplementary Information.
