# Supplementary material for: Autoregulation and dual stepping mode of MYA2, an Arabidopsis myosin XI responsible for cytoplasmic streaming
Source: Sci Rep. 2022 Feb 24;12:3150. doi: 10.1038/s41598-022-07047-0 (PMC8873201; doi:10.1038/s41598-022-07047-0)
Supplement: Supplementary file 1 — Supplementary Information. [file 41598_2022_7047_MOESM1_ESM.pdf]

Supplementary materials

Table S1

	TMR label	QD label
Velocity<br>(nm s^{-1})	10 ± 5<br>(n = 94)	17 ± 6<br>(n = 185)
Run length<br>(mean ± s.e.; nm)	208 ± 21<br>(n = 94)	179 ± 16<br>(n = 185)

Run length and velocity at 500 nM ATP for TMR and QD labeling myosin XI.

Table S2

	Tobacco 175 kDa myosin XI*	MYA2
Velocity<br>(μm s^{-1})	4.6<br>(1 mM ATP, 20 ºC)	5.2<br>(3 mM ATP, 25 ºC)
Run length<br>(nm)	1,320<br>(1 mM ATP)<br><br>1,430<br>(1 μM ATP)	non-processive<br>(1 mM ATP)<br><br>208<br>(500 nM ATP)
Duty ratio	0.81	0.32
Force<br>(pN)	0.5	0.85
Step size<br>(nm)	35<br>(1 mM and 1 μM 100 ATP; optical trapping assay)	32<br>(1 μM ATP; optical trapping assay)<br><br>29 and 60<br>(1 μM ATP; FIONA)

Comparison of molecular motile properties of tobacco 175 kDa myosin XI and MYA2.

* Tominaga, M. et al. Higher plant myosin XI moves processively on actin with 35 nm steps at high velocity. *EMBO J.* **22**, 1263-1272 (2003).

Figure S1

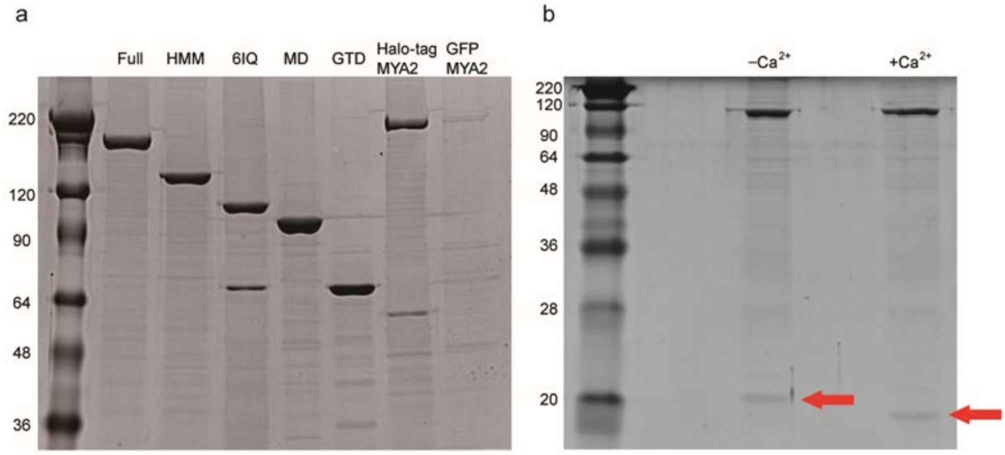

SDS-PAGE analysis of purified myosins. (a) Purified MYA2 Full, HMM, 6IQ, MD, GTD, Halo-tag MYA2, and GFP-MYA2 were analyzed using 8% SDS-PAGE and stained with Coomassie Brilliant Blue. The positions of molecular mass markers are indicated on the left (kDa). (b) Purified 6IQ was electrophoresed on 14% SDS-PAGE gel and stained with Coomassie Brilliant Blue. 6IQ was supplemented with 10 mM EGTA (− Ca^{2+}) or 10 mM $CaCl_2$ (+ Ca^{2+}). Low molecular mass bands of 6IQ (indicated by the red arrow) shift the mobility by Ca^{2+}, typical of calmodulin. The positions of molecular mass markers are indicated on the left (kDa).

Figure S2

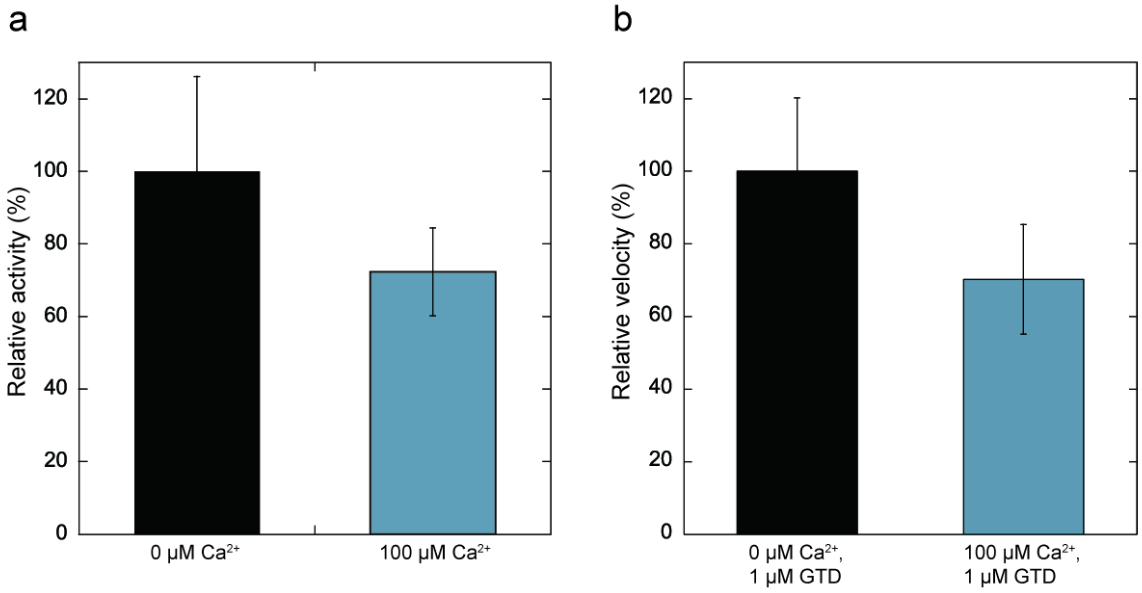

Effect of Ca^{2+} on the inhibitory effect of GTD on ATPase activity and motility. (a) Effect of Ca^{2+} on Mg^{2+}-ATPase activities of Full in the presence of 11.9 μM actin. (b) Effect of Ca^{2+} on the motility of HMM in the presence of GTD.

Figure S3

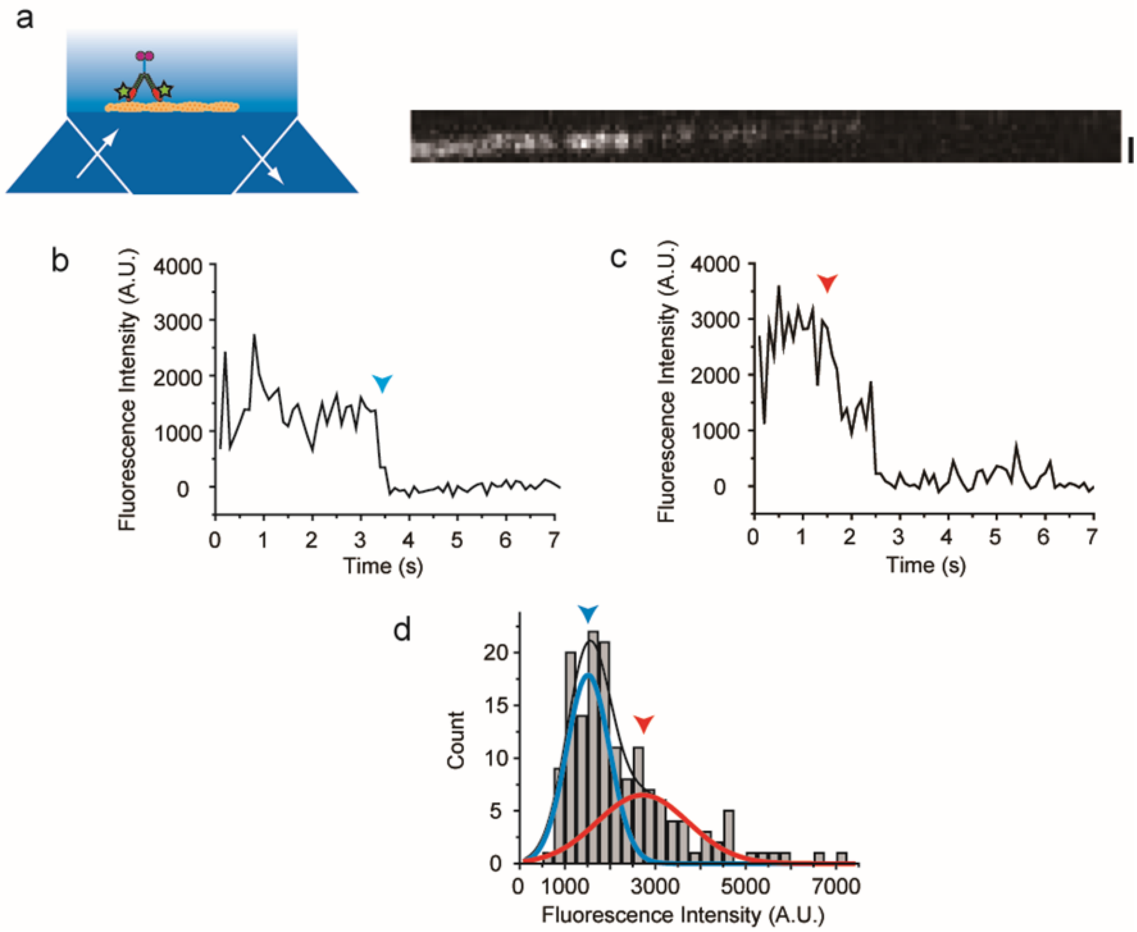

Properties of photo bleach reaction and fluorescent intensity of GFP-fused MYA2. Photo bleach reaction of the fluorescence of GFP-fused MYA2 processively moving was monitored using TIRFM with resolution of 0.1 second time. (a) Schematic drawing of single-molecule imaging (left) and a typical kymograph showing a processive motion of GFP-fused MYA2 (right). Scale bar, 500 nm. Single-step (b) or double-step (c) photo bleach reaction was observed. The blue and red arrowhead indicates the first photo bleach reaction. (d) The histogram of mean fluorescence intensity before the first photo bleach. The histogram was fit to two Gaussian functions (1,500 ± 300 (mean ± s.d.) for blue arrowhead and 2,700 ± 600 (mean ± s.d.) for red arrowhead, respectively).

Figure S4

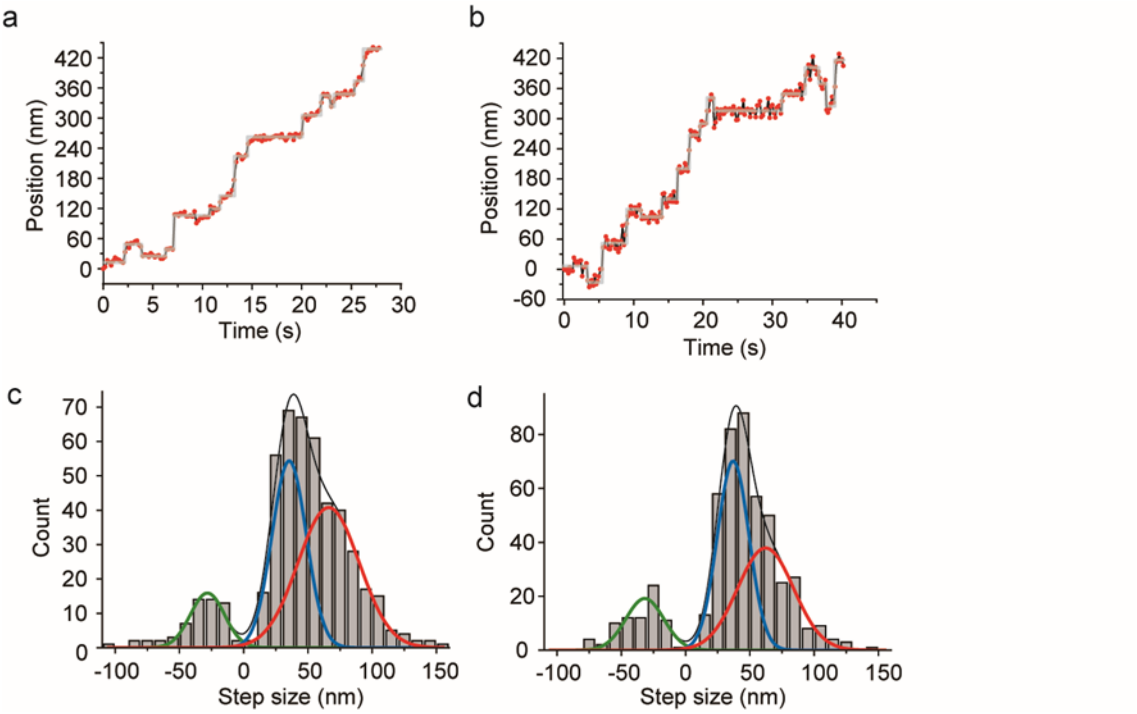

Stepping behavior of a MD in MYA2 dimer labeled with QD or TMR. (a and b) Typical stepping motion of QD-labeled MYA2 (a) and TMR-labeled MYA2 (b). TMR-labeled MYA2 was observed with 0.5 second time resolution. Steps were analyzed by an automated step-finding algorithm (gray line). (c) Histogram of step sizes of QD-labeled MYA2. (d) Histogram of step sizes of TMR-labeled MYA2. The histogram was fitted by three Gaussian functions peaks at 65 ± 17 nm, 35 ± 9.3 nm, −28 ± 9.2 nm for (c) and 62 ± 15 nm, 37 ± 8.7 nm, and −31 ± 10 nm for (d), respectively.

ATP concentration, 500 nM.

Figure S5

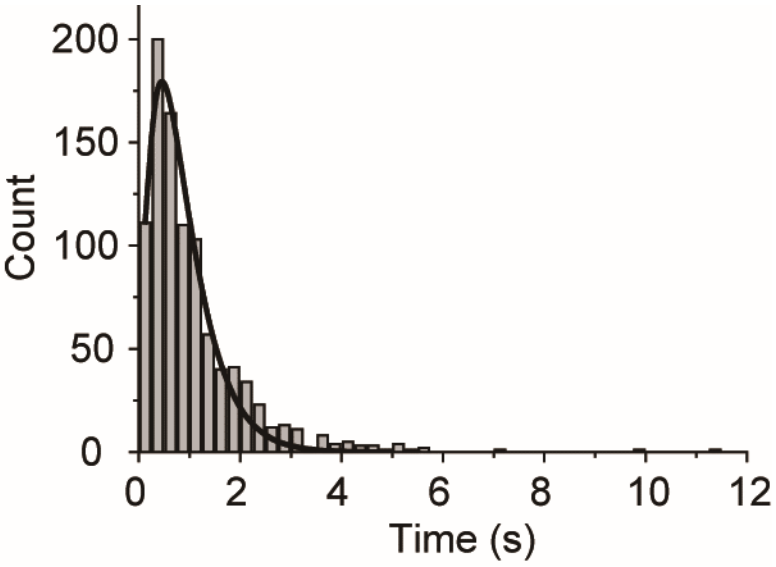

Distribution of dwell times between steps observed using TIRFM and single-molecule imaging and optical trapping assay. Histogram of the dwell time for forward steps observed by QD-labeled MYA2 at 1μM ATP. The histogram was fit to a convolution of two exponentials ($tk^2\exp(-kt)$), each having the same rate constant, *k* (2.5 s^{-1}).

Figure S6

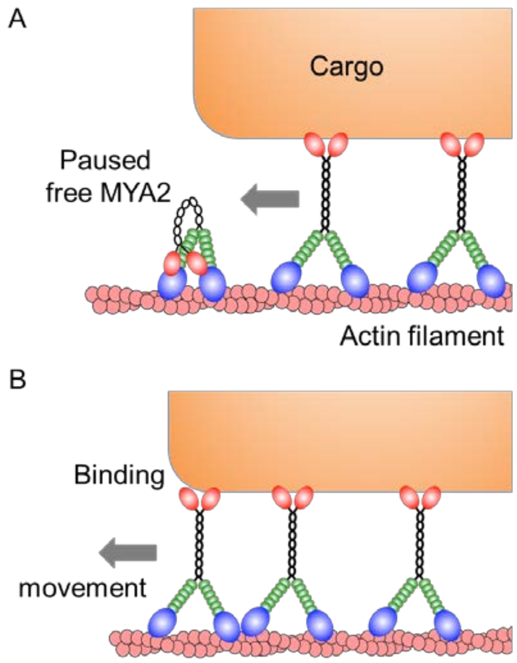

Transport regulation model by GTD inhibition *in vivo*. A. When MYA2 is not bound to cargo, it would pause on the actin filament while suppressing ATP consumption. B. As soon as the GTD binds to a new cargo, MYA2 can participate in the transport.
